# Realistic 3D morphology reshapes insect heat budgets

**DOI:** 10.1038/s41598-026-40212-3

**Published:** 2026-05-13

**Authors:** Madeleine M. Ostwald, Meredith G. Johnson, Abigail Youngblood, Alexis Childress, Katja C. Seltmann

**Affiliations:** 1 School of Biological and Behavioural Sciences, Queen Mary University of London, London, UK; 2 Cheadle Center for Biodiversity and Ecological Restoration, University of California, Santa Barbara, CA USA; 3 Department of Biological Sciences, North Dakota State University, Fargo, ND USA

**Keywords:** Heat balance, Thermal performance, Thermal physiology, Morphological scaling, Allometry, *Apis mellifera*, Ecology, Ecology, Zoology

## Abstract

Modeling insect heat exchange and predicting thermal responses depends on accurate representation of body size and shape. Still, most biophysical models approximate these complex forms using simplified geometric solids, whose relationships to real body forms have not been rigorously tested. Advances in surface modeling of small objects allow us to interrogate these assumptions by capturing the real 3D complexity of insect body forms. We used photogrammetry to construct 3D models of honey bee specimens and empirically measured body volume and surface area. Compared to empirical measurements, we found that traditional, geometric size estimation methods systematically underestimate body surface area and volume. We incorporated these error estimates into published heat budget data and found that these errors propagated non-linearly through the model, shifting the relative dominance of convective and radiative heat loss as temperature increases. These results suggest that body size and surface area assumptions can distort modeled heat transfer, particularly under low temperatures, demonstrating that morphological simplifications can bias physiological inference. This work underscores the utility of empirical 3D morphology for refining biophysical models of insect thermoregulation.

## Introduction

An organism’s thermal biology is determined in part by its size and shape^1^. For ectotherms, in particular, body surface area and volume mediate the exchange of heat with the environment^2,3^. Small-bodied organisms like insects have high surface area-to-volume ratios, and thus experience accelerated heat loss over their proportionally larger body surface^2,4^. This relationship is also influenced by morphology, with more elongated body forms having higher proportional surface area than more compact forms^5,6^. Quantifying these relationships between morphology and thermal biology is essential for predicting differential responses to climate conditions across phenotypically diverse taxa.

Biophysical models provide useful frameworks for linking morphology to thermal performance under varying environmental conditions^7–11^. Heat budgets, for example, are biophysical models that estimate rates of heat transfer attributable to environmental sources (e.g., radiation, conduction, convection) and internal physiological processes (e.g., metabolic heat production, evaporative cooling). Body size and morphology are key parameters in these models because they directly influence rates of heat loss and gain. Specifically, body surface area represents the interface through which insects interact with the thermal environment, regulating the degree of radiative heat gain, convective heat loss to moving air, conductive heat exchange, and evaporative heat loss. Relatedly, body volume and mass determine an individual’s thermal inertia, or the degree of energy required to change body temperature.

Consequently, biophysical models depend on our ability to accurately quantify insect size and form. Traditionally, bee body surface area and volume have been quantified indirectly by conceptualizing body segments (tagmata) as ideal solid shapes, for example, by assuming the thorax represents a sphere of a given diameter and estimating surface area and volume from geometric equations^12–14^. These methods provide useful alternatives to empirical measurements, which have been hindered by practical difficulties of measuring small, fragile, and complex forms. As a result, these methods have not yet been empirically validated; the error in geometric size estimates is unknown. Recent advancements in 3D surface modeling of small objects at relatively low cost place these empirical measurements within reach^2,15,16^. One such technique, photogrammetry, reconstructs a 3D model from a series of 2D images of an object taken from multiple angles. This approach has been validated in biological systems, including insects, as providing accurate size representation and reliably capturing external form at high resolution^17–22^. These methods may represent important improvements over traditional geometric estimates that ignore complex variation in 3D morphology.

We asked how honey bee (*Apis mellifera* Linnaeus 1758) size measurements from 3D models differ from those estimated from geometric equations, and what the implications of these differences are for downstream biophysical modelling. Honey bees are a classic model system for understanding mechanisms of thermoregulation insects^9,12,14,23,24^. We used photogrammetry to construct 3D models of honey bee specimens, from which we collected empirical measurements of body surface area and volume. We compared these measurements to geometric estimates from linear measurements of the same specimens, to estimate the percent error in these estimation methods. Finally, we incorporated our error estimates into a biophysical model using published data for honey bees in flight^14^, to understand how size error influences estimates of heat transfer across a realistic temperature range. Together, these results clarify the consequences of size assumptions for understanding routes of heat exchange in these ecologically important pollinators.

## Methods

### Specimens, photogrammetry, and 3D modeling

We measured body size of 11 *Apis mellifera* worker specimens housed in the University of California, Santa Barbara Invertebrate Zoology Collections. These specimens were collected in southern California (Santa Barbara County and Ventura County) between 1982 and 2019. Full specimen data is available at 10.5281/zenodo.17599627.

We constructed 3D models of all 11 worker honey bee specimens using techniques in photogrammetry^25^; (Fig. 2). We photographed specimens using a Macropod Pro 3D imaging system (Macroscopic Solutions, LLC, East Hartford, CT, USA) comprised of a Canon EOS 6D Mark II camera with a Canon EF 100 Macro USM AF/MF Lens mounted on a tripod and attached to a stage for automated specimen rotation. We pinned specimens to a stage and tilted them at a 15° angle so that both the ventral and dorsal surfaces of the specimen were captured in the images. We rotated bees 360°, taking an image every 2.6° of rotation, using a Stackshot macrorail with a 0.952 mm step size across the entire focal plane of the bee. We assembled photos at each angle using Zerene Stacker Software and then modified photos in Adobe Photoshop to improve image contrast. We used these photogrammetric image suites to construct 3D models of each specimen in Agisoft Metashape Pro Software (version 2.1, Agisoft LLC, St. Petersburg, Russia). All models and associated images are available at 10.5281/zenodo.17823482, including additional models for 2 queen honey bees and 6 drones, which were not included in the analysis. A detailed 3D modeling protocol is available at https://zenodo.org/records/17956682.

To ensure we accurately measured the size and shape of the underlying body, we manually removed areas of excess hair from 3D models. Photogrammetry surface reconstructions capture the external envelope of the bee’s body rather than resolving individual hairs. While bee hair may influence both surface reconstruction and heat transfer, its primary biophysical role is likely through modification of boundary-layer properties rather than through direct contributions to geometric surface area^26^. Importantly, body temperatures, metabolic rate, and evaporative water loss were measured empirically in Roberts and Harrison^14^, meaning that the net thermal effects of hair are implicitly incorporated into our heat budget calculations.

### Surface area and volume measurements

**Fig. 1:**
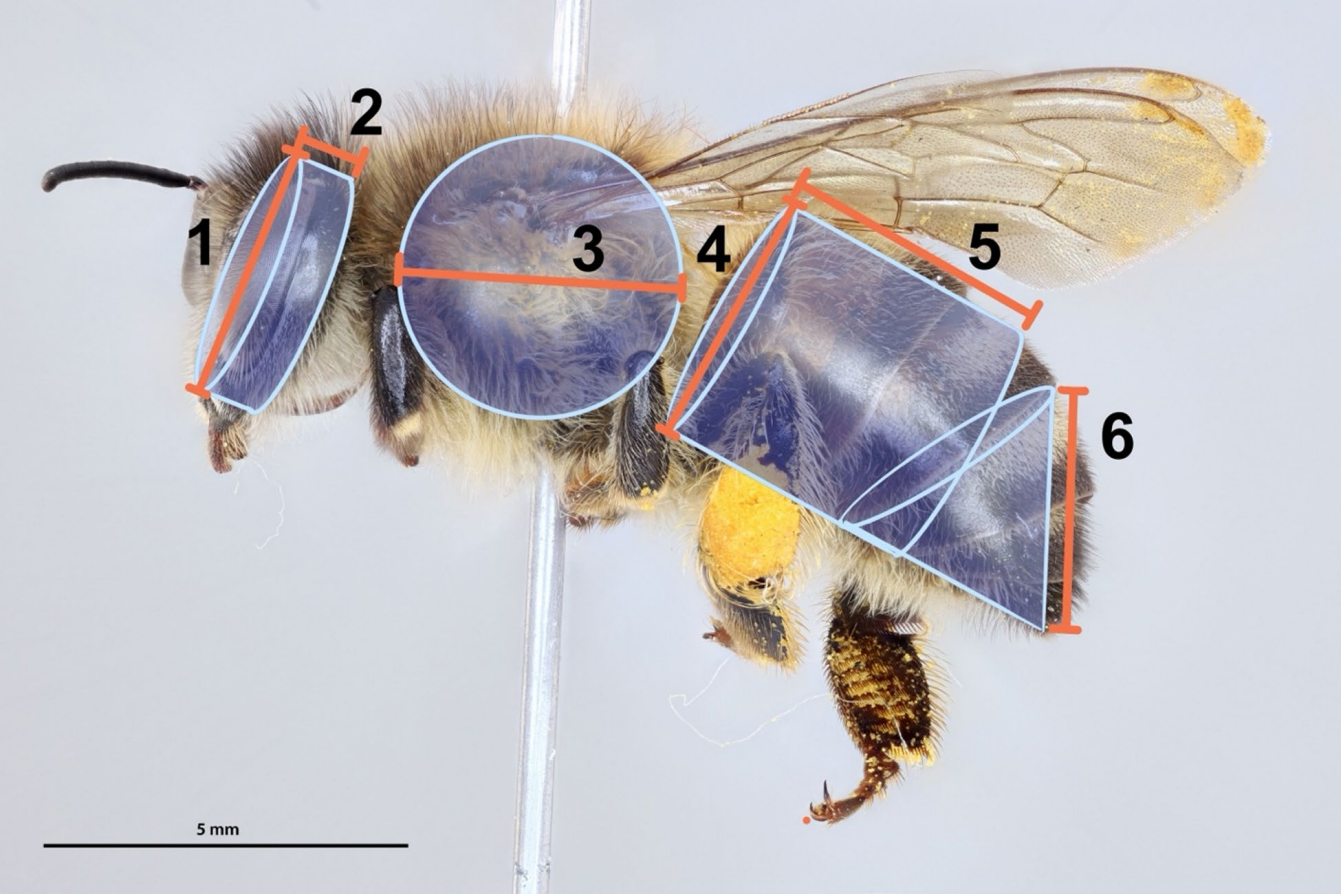
Diagram showing methods for size estimation via geometric equations. The head, mesosoma, and metasoma are assumed to be a cylinder, sphere, and cylinder + cone, respectively. Their volumes and surface areas are estimated from geometric equations for these solid shapes. The measurements used for calculation come from the following morphometric measurements: (1) the width of the head at the widest point, (2) the thickness of the head at the thickest point, (3) the width of the mesosoma at the widest point, (4) the width of the metasoma at the widest point, (5) the distance from the proximal end of tergite 1to the distal end tergite 3, and (6) the distance from the proximal end of tergite 4 to the distal end of tergite 6 for females or tergite 7 for males.

We measured surface area and volume of all specimens according to two methodologies: traditional estimates using geometric equations and morphometric measurements of the specimens themselves, and empirical measurements from 3D models. For the geometric estimates, we conducted the following specimen measurements using digital calipers (precision = 0.01 mm): (1) the width of the head at the widest point, (2) the thickness of the head at the thickest point, (3) the lateral width of the thorax at the widest point, (4) the width of the abdomen at the widest point, (5) the distance from the proximal end of tergite 1to the distal end tergite 3, and (6) the distance from the proximal end of tergite 4 to the distal end of tergite 6 for females or tergite 7 for males (Fig. 1). To estimate body size, we assumed the head was a cylinder, the mesosoma was a sphere, and the metasoma was a cylinder and a cone, then used the geometric equations for the surface area and volume of these solid shapes, in line with traditional methods^7,13,14^.

**Fig. 2:**
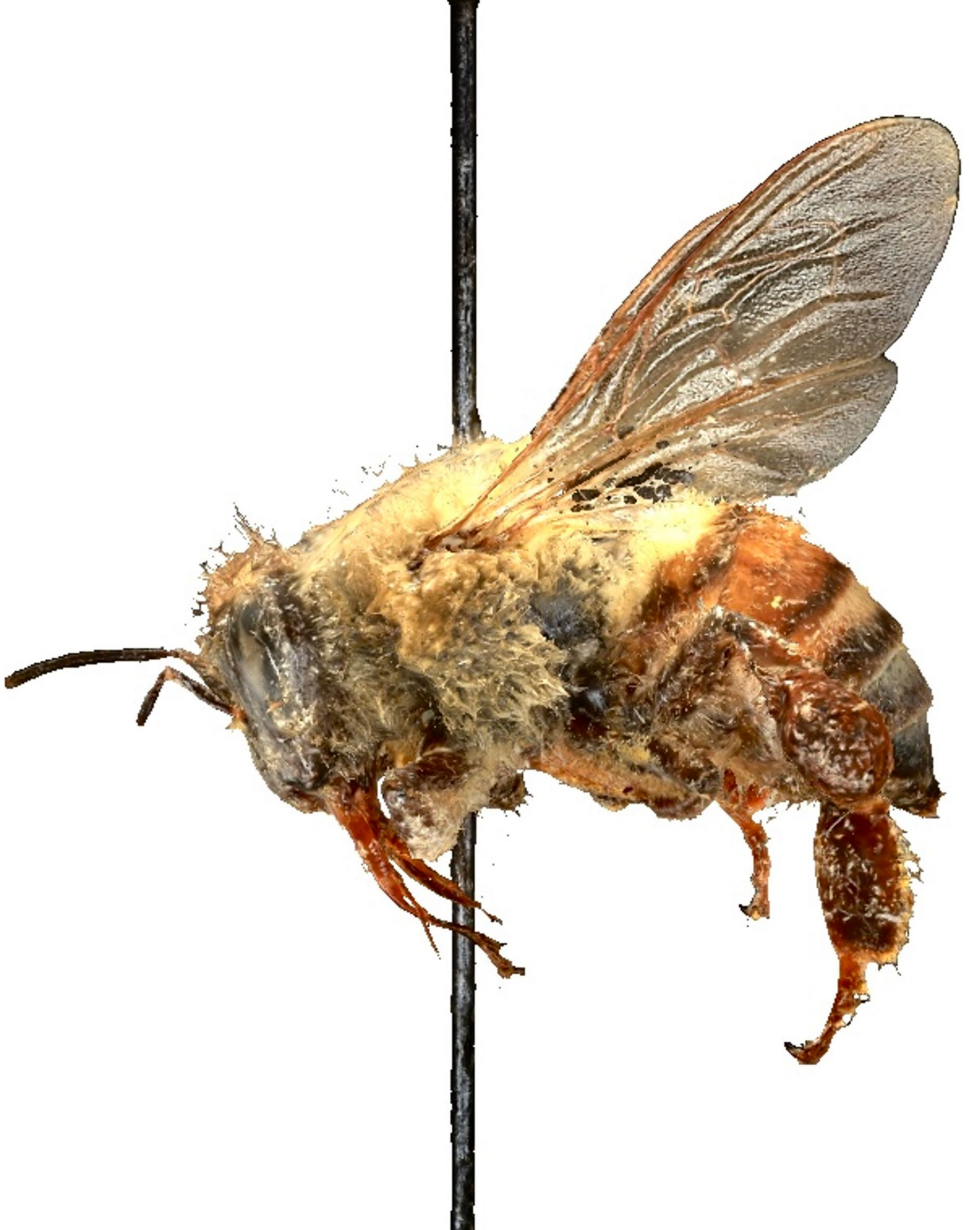
Photogrammetric 3D model of a single worker honey bee specimen.

We also measured surface area and volume of body tagmata (*i.e.*, head, mesosoma, metasoma) from 3D models of each specimen. We calibrated our measurements by imaging a scalebar with zoom and camera settings identical to those used for specimen imaging. The calibrated scale is then verified in 3D using a series of three known measurements taken from the models. From our tagma measurements, we excluded all other body parts (wings, legs, tongues, stingers, and antennae). Finally, we also measured the surface area and volume of the entire bee, including wings and legs (Fig. 2).

### Heat budget calculations

To approximate a honey bee heat budget at steady state (where heat gain = heat loss) across air temperatures of 20 to 50 °C, we used models generated from data reported in Roberts and Harrison, 1999. We calculated our heat budget as:

1 $$Q_{M} + Q_{E} + Q_{R} + Q_{C} = \, 0$$

where *Q*_M_ is metabolic heat production (mW) and *Q*_E_ is evaporative heat loss (mW) calculated from rates of CO_2_ and H_2_O production during flight in a closed system metabolic chamber. For full experimental details that include chamber set up, gas sampling, and calibration, please refer to Roberts and Harrison (1999). *Q*_R_ (mW) is longwave radiative heat transfer, and *Q*_C_ (mW) is convective heat transfer back-calculated as:

2 $$Q_{C} = \, \left( { - Q_{M} {-}Q_{E} {-}Q_{R} } \right)$$

To approximate *Q*_M_ and *Q*_E_, we used equations of *Q*_M_ and *Q*_E_ on air temperature to predict each route of heat transfer at 20, 25, 30, 35, 40, 45, and 50 °C. *Q*_M_ and *Q*_E_ were presented in mass-specific units of mW∙g^-1^, so we multiplied each value by the mean body mass of bees used in Roberts and Harrison, 1999 (75.3 mg) to determine absolute values of heat transfer for the average honey bee worker. *Q*_M_ and *Q*_E_ implicitly incorporate bees’ thermoregulatory responses expressed under experimental conditions. Consequently, our heat budget reflects the realized steady-state heat balance achieved by the average worker at each air temperature.

To calculate longwave radiative heat transfer, we used equations of head, thorax, and abdomen temperature as a function of air temperature to predict tagma temperature at 20, 25, 30, 35, 40, 45, and 50 °C ^14^. We used mean surface areas of honey bee head, thorax, and abdomen calculated using geometrical assumptions. We determined net longwave radiative heat transfer (*Q*_R_) for each tagma by subtracting radiative loss from radiative gain (*R*_gain_ – *R*_loss_).

3 $$\left( {R_{gain} {-}R_{loss} } \right)_{tagma} = \, \left[ {\left( {a \cdot \varepsilon_{C} \cdot \sigma \cdot T_{i}^{4} } \right) \, {-} \, \left( {\varepsilon_{S} \cdot \sigma \cdot T_{x}^{4} } \right)} \right] \, \cdot SA_{tagma}$$

where a is body surface absorptivity (0.98); ε_C_ and ε_S_ are emissivity of the borosilicate glass chamber where metabolic and evaporative water losses were measured (0.90) and emissivity of the bee’s body surface (0.98); σ is the Stefan-Boltzmann constant (5.67e10^–8^ W ∙ m^-2^ ∙ K^-4^); and *T*_i_ and *T*_x_ are the temperatures of the glass chamber walls assumed to be the same as air temperature and temperature of the bee’s body surface assumed to be the same as internal temperature^27^. We separately calculated net longwave radiative heat transfer for each tagma [(*R*_gain_—*R*_loss_)_tagma_] and multiplied by tagma surface area (*SA*_tagma_). We summed radiative heat exchange for each tagma to determine whole body radiative exchange.

In addition to the heat budget calculated using geometrical assumptions of surface area, we recalculated heat budgets based on the percent error of each tagma surface area measurement (Eq. 4; Table 1). We assumed that geometrical measurements (*SA*_geometric_) underestimated theoretical surface area (*SA*_theoretical_) based on our 3D models.

4 $$SA_{theoretical} = SA_{geometric} \cdot \, \left( {1 \, {-} \, percent \, error} \right)^{ - 1}$$

**Table 1:** Summarized results of the statistical comparisons between geometric measurements of worker body regions and empirical measurements from 3D models (*N* = 11). Statistically significant differences are indicated with asterisks.

Body region	Measurement	Test statistic (*t*)	*P*-value	% error (mean ± std. error)	Mean signed error (MSE)
Head	volume	− 6.853	< 0.001 ***	39.298 ± 3.870	− 9.966
	surface area	− 6.286	< 0.001 ***	26.424 ± 3.390	− 14.303
Mesosoma	volume	− 6.589	< 0.001 ***	50.261 ± 4.499	− 34.681
	surface area	− 7.512	< 0.001 ***	44.056 ± 3.994	− 49.936
Metasoma	volume	− 1.249	0.240	NA	NA
	surface area	− 1.898	0.087	NA	NA
Head + Mesosoma + Metasoma	volume	− 3.576	0.005 **	39.471 ± 5.037	− 51.792
	surface area	− 3.846	0.003 **	15.202 ± 2.302	− 32.304

### Statistical analysis

We assessed whether geometric estimates of body size (surface area and volume) differed significantly from measurements taken from 3D models using paired *t*-tests. We verified assumptions of normality were met using diagnostic plots and Shapiro–Wilk tests. We conducted these tests for comparisons between the size (surface area and volume, separately) of the head, mesosoma, metasoma, and the sum of the three tagmata. Where measures differed significantly, we estimated the magnitude of this difference as the percent error for each estimate (head, metasoma, mesosoma, summed tagmata) as follows:

5 $$Percent Error = \left( {\frac{{\left| {Geometric Size Estimate - 3D Model Size Measurement} \right|}}{3D Model Size Measurement}} \right) \times 100$$

Finally, we asked whether geometric estimates tended to over- or under-estimate volume and surface area relative to 3D model measurements by calculating the mean signed error (MSE, the average of the signed differences between paired measurements) for each paired measurement group. Results are reported as mean ± standard error.

To examine how body surface area (*SA*) scales with body volume (*V*), we fit linear models to log-transformed data, where the slope represents the scaling exponent. Under geometric similarity, surface area is expected to scale with volume to the two-thirds power^28^. To test whether scaling exponents differed among surface area estimation methods (geometric, 3D model of head + mesosoma + metasoma, and whole body 3D model including wings and legs), we combined all three datasets and fit a linear model with an interaction term between log(volume) and estimation method. To test whether each slope significantly differed from the theoretical geometric expectation (2/3), we used the linearHypothesis() function in the *car* package^29^.

To determine how errors in surface area measurements propagate through heat budget calculations, we compared heat budgets calculated under two assumptions of surface area: (1) geometric estimates following Roberts and Harrison (1999) and (2) our 3D-derived estimate. For metabolic heat production and evaporative heat loss, we extracted modeled heat gains and losses across a biologically relevant range of air temperatures (20–50 °C) from Roberts and Harrison^14^. Using surface areas and tagma temperatures, we calculated longwave radiative transfer and convective heat transfer. We used linear regression models to quantify the relationship between air temperature and heat transfer (*Q*_X_) for each surface area assumption. For each route, we fit models of the form: [*Q*_X_] = intercept + [*T*_air_] + [Method] + [*T*_air_∙Method]. Method refers to the method of surface area measurement. The interaction term tested whether slopes differed among assumptions. Analysis of variance (ANOVA) was used to assess the significance of main effects and interactions. In instances where interaction terms were not significant, we interpreted differences among routes as shifts in intercept. All analyses were performed in R version 4.4.2^30^.

## Results

### Geometric equations underestimate bee body volume and surface area

**Fig. 3:**
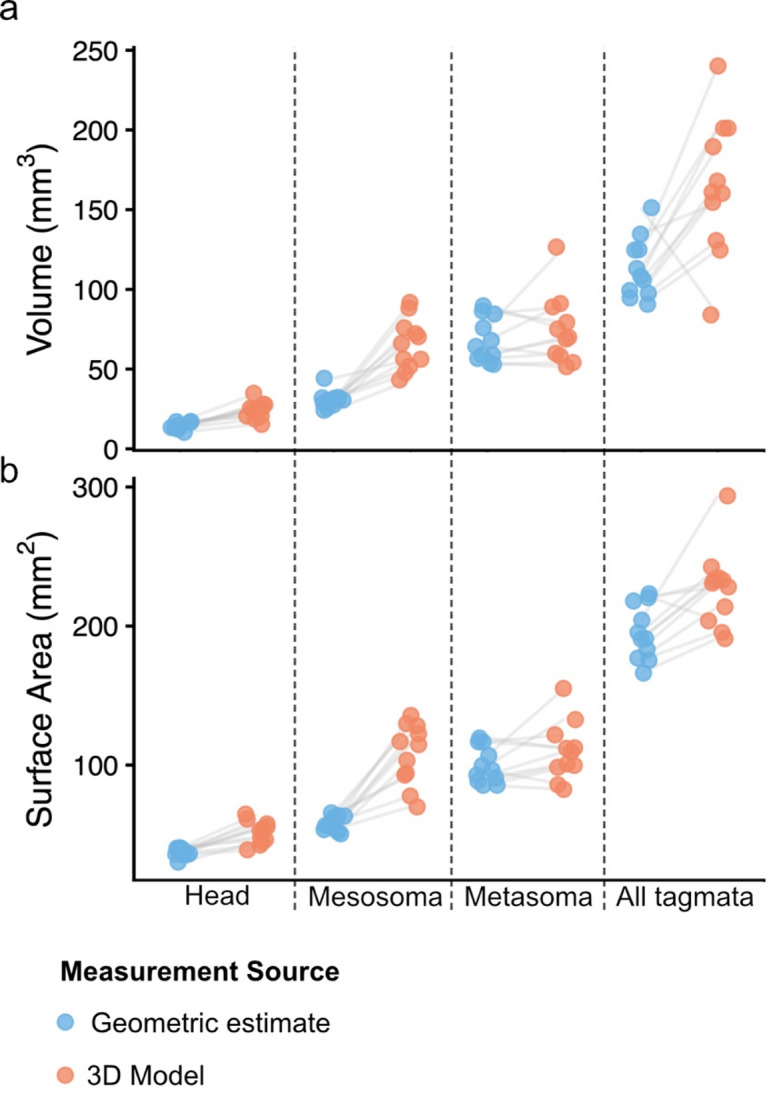
Surface area and volume measurements for worker heads, mesosomas, metasomas, and summed tagmata (head + mesosoma + metasoma), compared between geometric estimates (blue) and empirical measures from 3D models (orange). Gray lines indicated paired comparisons between measurement methods for the same specimen.

**Fig. 4:**
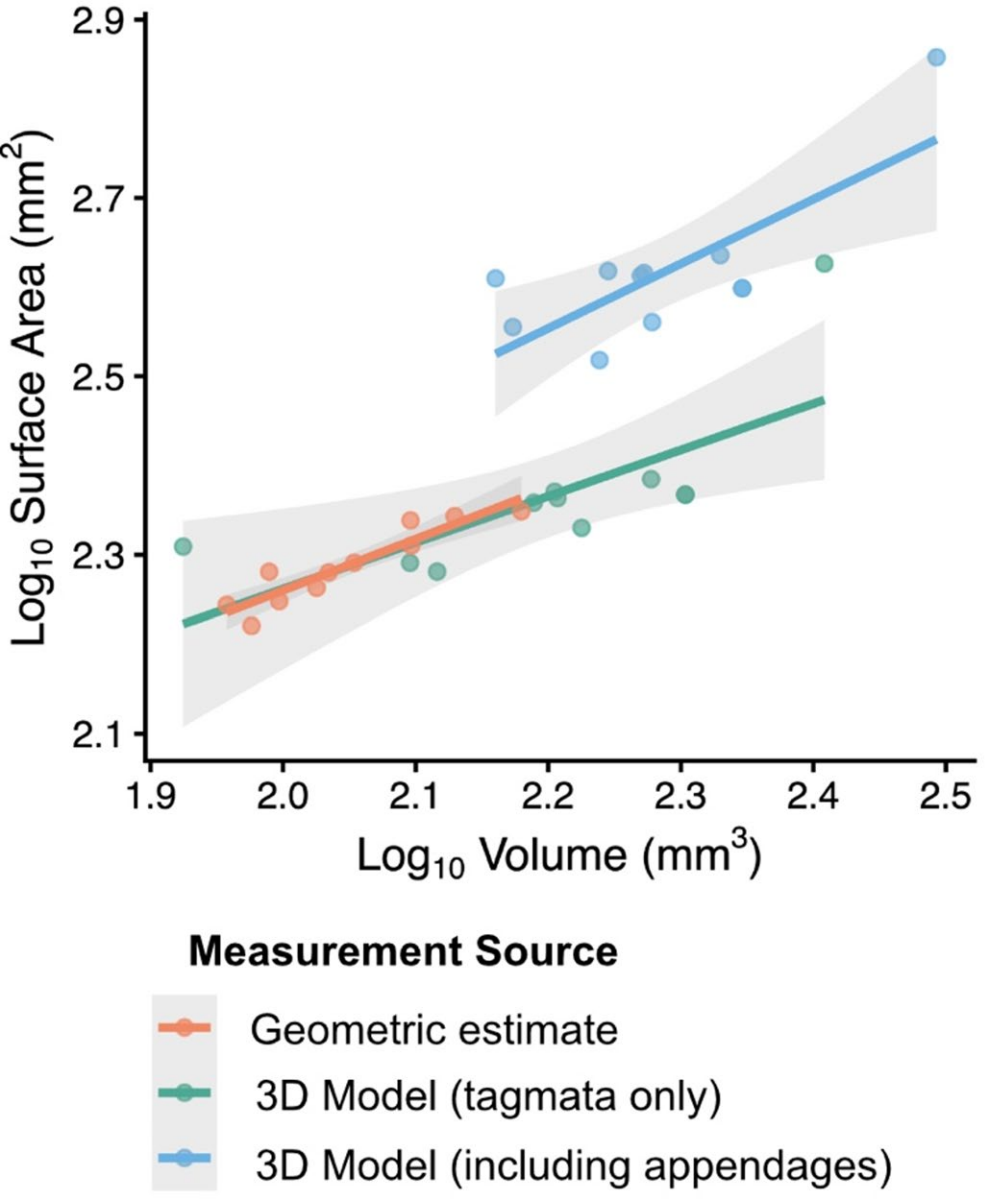
Allometric scaling of log_10_-transformed surface area and volume from geometric estimates (orange; *β* = 0.574 α = 1.112, *R*^2^ = 0.847, *P* < 0.001, 95% CI *β* (0.40, 0.75)), from 3D models of the summed head, mesosoma, and metasoma (green; *β* = 0.518, α = 1.224, *R*^2^ = 0.466, *P* = 0.012, 95% CI *β* (0.14, 0.90)), and from 3D models of the entire body, including legs and wings (blue; *β* = 0.723, α = 0.962, *R*^2^ = 0.541, *P* = 0.005, 95% CI *β* (0.27, 1.18)).

Geometric estimation methods significantly underestimated surface area and volume of worker head, mesosoma, and summed tagmata (head + mesosoma + metasoma) relative to measures taken from 3D models (Table 1; Fig. 4). Geometric estimates of head volume and surface area underestimated model measures with 39.298 ± 3.870% error and 26.424 ± 3.390% error, respectively (Paired *t*-tests: *P* < 0.001). Similarly, geometric estimates of mesosoma volume and surface area underestimated model measurements with 50.261 ± 4.499% error and 44.056 ± 3.994% error, respectively (Paired *t*-tests: *P* < 0.001). Geometric estimates of metasoma volume and surface area, however, did not differ significantly from model estimates (Paired *t*-test: *P* = 0.240; Paired Wilcoxon test: *P* = 0.087, respectively). The summed tagmata represented by the head, mesosoma, and metasoma was significantly lower in geometric estimates of volume (Paired *t*-test: *P* = 0.006, 39.471 ± 5.037% error) and surface area (Paired *t*-test: *P* = 0.005, 15.202 ± 2.302% error). Including the wings and legs in model estimates increased the volume by 16.272 ± 3.622%, and the surface area by 43.627 ± 1.082% (Fig. 3).

Queens and drones, though not included in our statistical analysis, appeared to follow a similar trend of underestimation in geometric estimates of the head, mesosoma, and summed body tagmata (Fig. 4).

### Body surface area scales predictably with volume across measurement methods

We fit linear models to examine allometric scaling of log_10_-transformed surface area with volume for three measurement approaches: geometric estimates (*β* = 0.574 α = 1.112, *R*^2^ = 0.847, *P* < 0.001, 95% CI *β* (0.40, 0.75)), 3D models of the summed head, mesosoma, and metasoma (*β* = 0.518, α = 1.224, *R*^2^ = 0.466, *P* = 0.012, 95% CI *β* (0.14, 0.90)), from 3D models of the entire body, including legs and wings (*β* = 0.723, α = 0.962, *R*^2^ = 0.541, *P* = 0.005, 95% CI *β* (0.27, 1.18)). All models showed strong positive scaling with volume (Fig. 4). An interaction model testing whether slopes differed among methods found no significant interaction terms (*P* > 0.62), indicating that the scaling exponent did not differ statistically among methods. We tested whether observed slopes differed from the theoretical isometric expectation of 2/3 (0.667). Linear hypothesis tests did not reject the null for any method (all *P* > 0.25), indicating that the scaling exponents are statistically consistent with isometry.

### Size errors propagate through heat budget calculations

**Fig. 5:**
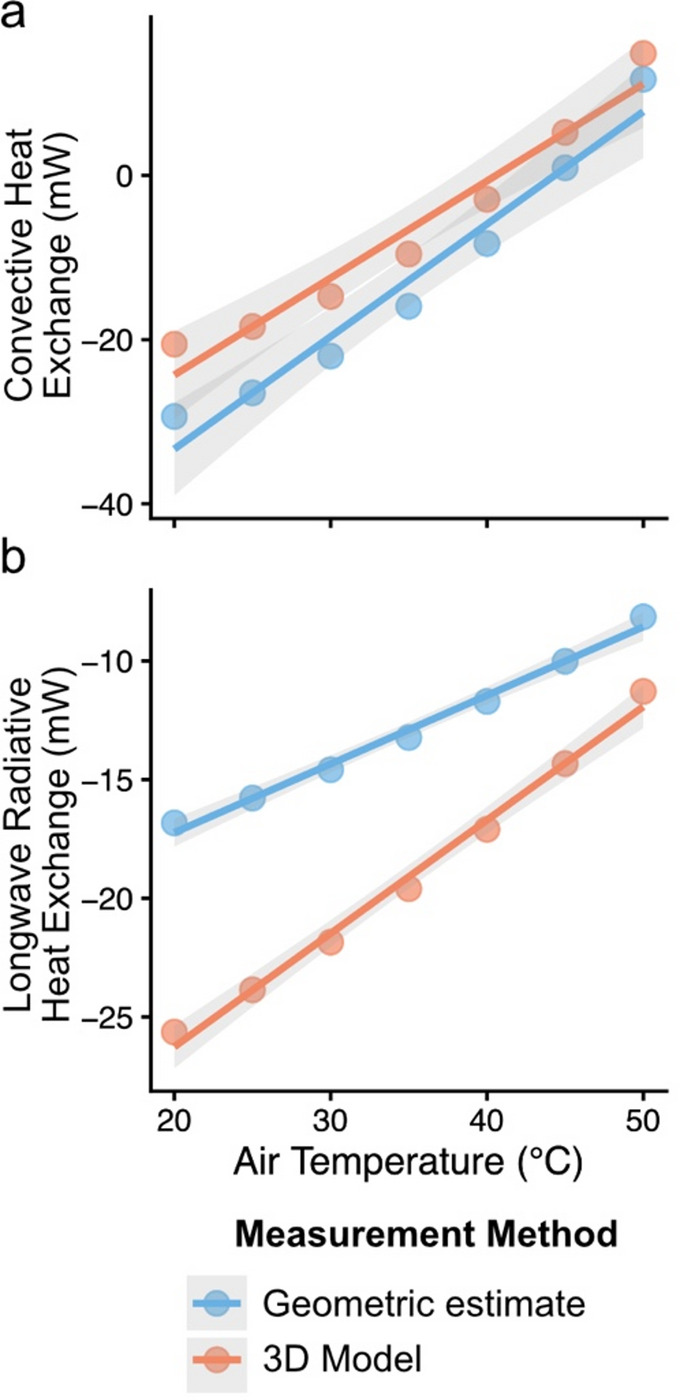
Effects of surface area estimation methods on (a) modeled longwave radiative exchange (*Q*_R_) and (b) convective heat exchange (*Q*_C_) across air temperatures. For longwave radiative exchange, the linear model showed a significant interaction between air temperature and route (F_1,10_ = 58.49, *P* < 0.001), indicating slopes differed between measurement methods (*β*_geometric_ = 0.289, α_geometric_ = − 23.01; *β*_3Dmodel_ = 0.465, α_3Dmodel_ = − 34.97). For convective heat exchange, the linear model showed no significant interaction between air temperature and measurement method (F_1,10_ = 1.09, *P* = 0.32), indicating slopes did not differ among measurement methods (*β*_geometric_ = 1.370; *β*_3Dmodel_ = 1.194). However, regression intercepts differed significantly between measurement methods (F₂,₁₅ = 13.5, *P* < 0.001); α_geometric_ = − 60.72; α_3Dmodel_ =− 48.77).

Underestimation of tagma surface area (Table 1) significantly altered heat budget estimates. For longwave radiation, the effect of temperature was significantly greater when we estimated surface area from 3D models (*β* = 0.465, 95% CI: 0.429–0.501) compared to geometric estimates (*β* = 0.289, 95% CI: 0.252–0.325; difference = 0.176, *P* < 0.001), indicating that geometric surface area estimation methods underestimate the increase in *Q*_R_ with temperature (Fig. 5a). In contrast, for convective heat loss, slopes did not differ significantly between models (geometric *β* = 1.370, 3D *β* = 1.194, *P* = 0.32), suggesting a consistent temperature response. However, intercepts differed, reflecting systematic shifts in absolute values depending on surface area estimation method (Fig. 5b).

**Fig. 6:**
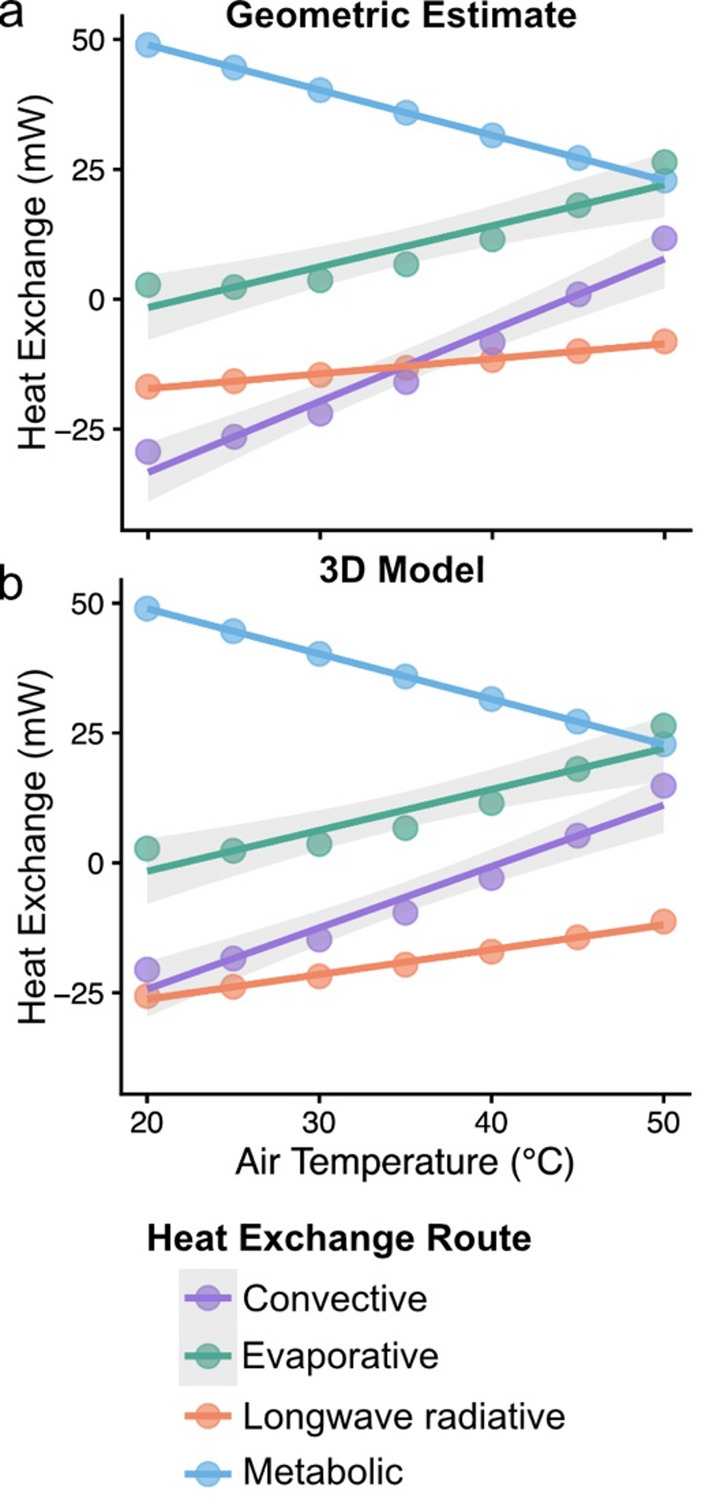
Heat budget estimates for (**a**) traditional geometric estimates (taken from Roberts and Harrison, 1999) and (**b**) expected empirical measures based on known percent error between geometric and 3D model approaches. Lines represent modeled contributions of convective heat exchange (purple), evaporative heat exchange (green), longwave radiative heat exchange (orange), and metabolic heat production (blue) to total heat exchange. For geometric estimates (a), longwave and convective heat exchange intersect near 35 °C, with convection dominating at lower temperatures and longwave radiation at higher temperatures. For 3D estimates (b), longwave radiation dominates across the full temperature range.

When considering complete heat budgets (including metabolic heat production, evaporative cooling, convective heat loss, and longwave radiative heat gains and losses), the dominant route of heat loss differed depending on the surface area estimation method used. For geometric estimates, longwave radiative and convective heat loss contributions intersected near 35 °C, with convection dominating at lower temperatures and longwave radiation at higher temperatures (Fig. 6a). In contrast, for 3D model estimates, longwave radiation consistently dominated across the full temperature range (Fig. 6b). Thus, geometric methods may misrepresent the relative importance of alternative heat loss pathways, particularly at low temperatures.

## Discussion

Insect body forms are complex and highly diverse, with correspondingly complex consequences for heat balance. Simplification of this complexity allows us to make reasonable predictions about thermoregulatory mechanisms in different environmental contexts. However, interrogating these simplifications enables us to improve our predictions to better resemble living systems. By comparing traditional, geometric methods for estimating bee body size to empirical measurements taken from 3D models, we demonstrate that geometric methods systematically underestimate honey bee body volume and surface area. We found that these underestimates propagate nonlinearly through biophysical models, altering both the magnitude and relative ranking of heat exchange pathways.

We found that geometric estimation methods significantly underestimate the surface area and volume of the worker head, mesosoma, and whole body relative to 3D model measurements. These underestimations of surface area propagate through heat budget calculations, resulting in systematic shifts in predicted heat gains and losses. For longwave radiation, geometric estimation of surface area reduced the absolute magnitude of heat loss and also flattened the slope of the relationship with air temperature, underpredicting the rate at which bees dissipate heat under increasing temperatures (Fig. 3A). A flatter slope means that heat transfer changes more slowly as air temperature increases. In contrast, for convective heat loss, slopes remained consistent across estimation methods, indicating that temperature-dependent responses are robust; however, absolute values were lower for the geometric model, reflecting the direct effect of surface area on heat transfer magnitude (Fig. 3B). These findings highlight the importance of accurate surface area measurements in thermoregulatory modeling, particularly for radiation-dominated heat loss, and suggest that geometric approximations may misrepresent absolute thermal heat gains and losses.

Our analysis did not include solar radiation, a route of heat gain for which accurate surface area measurements are similarly critical. The empirical heat exchange data used in our calculations were collected in a shaded chamber in laboratory conditions, where direct solar radiation was absent. Unlike longwave or convective heat loss, solar radiation scales directly with the projected surface area exposed to sunlight. Consequently, underestimation of surface area would likely have an even greater impact on predicted solar heat gain, potentially amplifying the differences observed among models. Future work incorporating solar radiation heat gain will be essential to fully quantify the thermoregulatory consequences of surface area measurement errors.

A major advantage of empirical surface area measurements is the inclusion of body parts (e.g., wings, legs, antennae) that are typically excluded from size estimates due to measurement difficulty. With their relatively large surface areas, these body parts may contribute substantially to overall heat transfer, increasing body volume by approximately 16% and surface area by 44%, relative to tagma-only estimates. Our heat budget calculation focused on individual tagmata (head, mesosoma, metasoma) to standardize the comparison to geometric estimates. Interestingly, whole-body 3D measures showed the greatest variance but also the highest scaling exponent (0.72). Because these data include more total surface area (e.g., wings, antennae, limbs), they also introduce more biological variability. That the results were still broadly consistent across methods (3D and geometrical assumptions) suggests that the estimated scaling exponent is robust, even if the absolute surface area values differ.

Beyond honey bees, the 20,000 species of bees globally represent a remarkable array of body forms. Bees vary more than 30-fold in body length, and vary in shape from compact, rounded forms (e.g., *Bombus*) to slender and highly elongated (e.g., *Tetragonisca*). Non-*Apis* bees are poorly represented in biophysical models (but see^7,13^. Their size and shape diversity will have considerable impacts on heat transfer that have not yet been clarified. Honey bees, with their characteristically torpedo-shaped metasomas, may be better suited to traditional geometrical assumptions than other species. This unique metasoma form, which does reasonably approximate ideal geometric forms (cylinder + cone), may explain our finding that geometric estimates of metasoma volume and surface area did not differ significantly from empirical measurements, despite clear differences in head and mesosoma estimates. Nevertheless, across all tagmata, geometric estimates poorly represented real body volumes and surface areas. These errors may be compounded in other bee species with more distinct morphologies, highlighting the urgency of generating empirical surface measurements for diverse bee species.

3D surface modeling represents an important but neglected methodological advance in biophysical modeling. Along with other 3D reconstruction approaches (e.g., x-ray tomography, structured-light surface modeling) photogrammetry represents an established and valuable tool for size estimation^2,31,32^. However, an important consideration is the time cost of generating 3D models, which entails skilled photogrammetry and model assembly by trained users. Photogrammetry systems can also be costly, though low-cost, 3D-printed options are becoming increasingly accessible^15^. We emphasize open model sharing as a solution to these obstacles^33^. Shared 3D models (and their associated images) can serve as reference material for parameterizing future biophysical models using size-matched individuals. These measurements represent crucial advances in realistically modeling complex biophysical relationships between insects and a warming environment.

## Data Availability

All data and analysis code associated with this study is published at 10.5281/zenodo.17599627. The 3D models and associated images are published in the associated repository at 10.5281/zenodo.17823482.
